# Visual Analysis of Colorectal Cancer Immunotherapy: A Bibliometric Analysis From 2012 to 2021

**DOI:** 10.3389/fimmu.2022.843106

**Published:** 2022-03-31

**Authors:** Long Ma, Jixiang Ma, Muzhou Teng, Yumin Li

**Affiliations:** The Second Clinical Medical College of Lanzhou University, Department of General Surgery, Lanzhou University Second Hospital, Key Laboratory of Digestive System Tumors of Gansu Province, Lanzhou, China

**Keywords:** colorectal cancer, immunotherapy, bibliometric, CiteSpace, immune checkpoint, gut microbiota

## Abstract

An increasing number of studies have shown that immunotherapy serves a significant role in treating colorectal cancer (CRC) and has become a hotspot. However, few studies used the bibliometric method to analyze this field comprehensively. This study collected 1,899 records of CRC immunotherapy from 2012 to October 31, 2021, and used CiteSpace to analyze regions, institutions, journals, authors, and keywords to predict the latest trends in CRC immunotherapy research. The United States and China, contributing more than 60% of publications, were the main drivers in this field. Sun Yat-sen University was the most active institution, while the National Cancer Institute had the highest frequency of citations. Most publications were published in the *Journal for Immunotherapy of Cancer.* Adam E Snook was the most prolific writer, while Dung T. Le was the most commonly co-cited author. “T cell”, “MMI” and “PD-1blocked” were the most widely studied aspects of CRC immunotherapy. “Immune checkpoint inhibitor”, “combination therapy”, “drug therapy” and “liver metastases” were current research hotspots. “Tumor microenvironment”, “neutrophils”, “tumor-associated macrophages”, and “suppressor cell” have emerged as research hotspots in recent years. “Gut microbiota”, “nanoparticle” and “tumor mutational burden” as recently emerged frontiers of research that should be closely monitored.

## Introduction

CRC is one of the most common cancers in the world. According to statistics, there have 1.9 million new cases of CRC in 2020, and it is expected to reach 2.5 million in 2035 (1). Although the availability of screening has improved the 5-year survival rate for CRC, approximately 30% of patients present with metastatic disease at diagnosis and approximately 50% of non-metastatic patients eventually progress to metastatic disease (2). Over the past decade, increased understanding of CRC pathogenesis has led to the recognising that almost all CRCs displayed activation of the RAS/RAF/MEK signaling cascade. Targeting EGFR, HER2, KRAS, NRAS, or BRAF, which were linked to their activation, has resulted in a reduction in the proportion of CRC patients. However, due to the highly mutagenic and adaptable nature of tumor cells, drug-resistant clones will arise in the vast majority of cases (3, 4).

Ioannides and Whiteside first introduced the tumor microenvironment (TME), which refers to the local biological environment during tumor development, providing a scaffold and barrier for tumor cell growth and generating immune-exempt areas to provide a ‘breeding ground’ for tumors. TME helps tumor cells evade immune surveillance by providing an environment for tumor growth and suppressing local immune responses (5, 6). A balance is established between the tumor and the adaptive immune system, shaping each other (7). Immunotherapy, which aims to use the immune system to fight tumors, has shown a promising future in treating CRC.

On May 23, 2017, the FDA approved Pembrolizumab for use in adult or pediatric patients with unresectable or metastatic, MSI-H/dMMR solid tumors, including colorectal cancer. On July 31 of the same year, the FDA approved Nivolumab to treat patients aged 12 years or older with MSI-H/dMMR colorectal cancer (2). on July 10, 2018, the FDA approved the combination of Ipilimumab and Nivolumab to treat patients aged 12 years or older with MSI-H/dMMR colorectal cancer. on June 29, 2020, the FDA again approved Pembrolizumab for intravenous infusion for the first-line treatment of patients with unresectable or metastatic MSI-H/dMMR colorectal cancer and does not require concomitant use with chemotherapy. Pembrolizumab and Nivolumab are PD-1 inhibitors, and Ipilimumab is a CTLA-4 inhibitor. T cells express PD-1 receptors that bind to their ligands PD-L1 and PD-L2, inhibiting T cell proliferation and cytokine production. PD-1 ligands are upregulated in some tumor cells, and signaling through this pathway can inhibit immune surveillance of tumors by activated T cells. PD-1 inhibitors are monoclonal antibodies that bind to the PD-1 receptor, blocking the interaction of PD-1 with PDL1 and PD-L2 and relieving PD-1 pathway-mediated suppression of immune responses, including antitumor immune responses. In a homologous mouse tumor model, blockade of PD-1 activity inhibited tumor growth (8, 9). Ipilimumab is an anti-CTLA-4 antibody. CTLA-4 is expressed on the surface of T cells. CTLA-4 exerts its inhibitory effect during the T cell activation phase: early in the immune response, CTLA-4 competes with CD28 to bind to B7 on antigen-presenting cells, and when CTLA-4 signaling is stronger, T cells are inhibited, while when CD28 signaling is stronger, T cells are activated and continue to replicate and produce greater killing capacity. Ipilimumab inhibits the competitive binding of CTLA-4 to CD28, thus allowing T cells to multiply and increasing the ability to kill tumors (10, 11).

Immune checkpoint inhibitors (ICIs), modulating the interaction of T cells, antigens-presenting cells (APCs) and tumor cells to help unleash suppressed immune responses, emerged as a very effective therapy for patients with mCRC that is mismatch-repair-deficient (dMMR) or microsatellite instability-high (MSI-H) (termed dMMR/MSI-H mCRC). Due to the efficacious, stable and durable responses, pembrolizumab and nivolumab (with or without Ipilimumab) were approved by US Food and Drug Administration (FDA) to treat these patients. However, mCRC is characterized by insufficient mutated tumor antigen (12), thus the main challenge is to provide the benefit of immunotherapy for the vast majority of mCRC patients that are mismatch-repair-proficient (pMMR) or microsatellite-stable (MSS) or low microsatellite instability (MSI-L) (termed pMMR/MSS/MSI-L mCRC) (13).

Bibliometric analysis adopts the number of citations as a proxy measure of research quality, which is a useful tool for assessing the trend of research efforts statistically and qualitatively. It provides an objective assessment of the contributions of academic groups and individual researchers through a comprehensive analysis of the authors, countries, journals, citations and publication dates of selected articles, thus providing a method for understanding trends in specific fields and ranking academic groups and individuals (14). In addition, keywords that appear more frequently in the included articles and hot words that have emerged in recent years were analyzed to provide supporting evidence for future trends (15).

There was currently no similar analysis in CRC. This paper utilized bibliometric analysis to describe the literature related to CRC immunotherapy over the last decade to understand its features and forecast future research trends and hotspots. Therefore, to better capture the current status and trends in CRC immunotherapy research, the purposes of this study were to reveal present study trends, explore into possible research hotspots and guide researchers in their future work by using bibliometric methods to visualize references.

## Materials and Method

**Figure 1:**
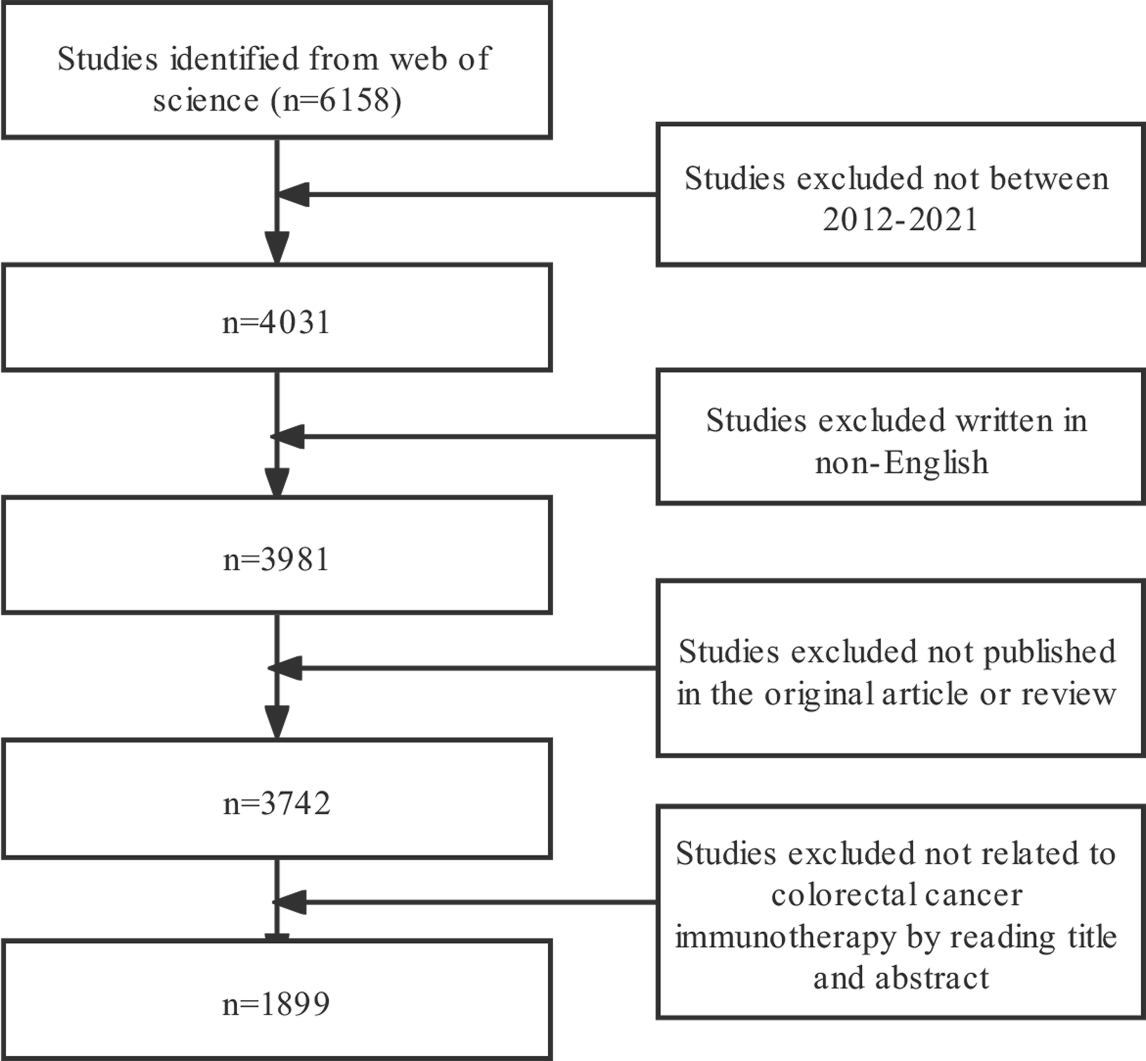
Flowchart depicting the article selection process.

The Web of Science (WOS) core database from Clarivate Analytics was deemed the best for bibliometric analysis (16, 17), so we selected it to perform the search. The WOS core database was searched on November 1, 2021, for all articles related to CRC immunotherapy from 2012 to October 31, 2021, using the following search formula: TS=(Rectal Neoplasm* OR Rectal Tumor* OR Rectal Cancer* OR Rectum Neoplasm* OR Rectum Cancer* OR Cancer of the Rectum OR Cancer of Rectum OR Colorectal Neoplasm* OR Colorectal Tumor* OR Colorectal Cancer* OR Colorectal Carcinoma* OR Colonic Neoplasm* OR Colon Neoplasm* OR Cancer of Colon OR Colon Cancer* OR Cancer of the Colon OR Colonic Cancer*) AND TS=(Immunotherapy OR Immunotherapies OR immunotherapeutic). The literature inclusion criteria were as follows: (1) The manuscript was based on the theme of immunotherapy for CRC and the whole content was accessible; (2) Document types included Article and Review;(3) Written in English. The exclusion criteria were as follows:(1) The major themes were not related to immunotherapy for CRC or could not be evaluated. (2) Articles were meeting abstracts, news, briefings, etc. The complete text was evaluated by two reviewers separately against the inclusion and exclusion criteria. Any disagreements between the two reviewers (L M and JX M) were resolved by negotiation and the document was exported in plain text format (**Figure 1**).

The annual publication was analyzed and plotted using GraphPad prism v8.0.2. Furthermore, bibliometric methods were used to gather the research foundation, frontier knowledge, and trends. In this study, the acquired bases were imported into the CiteSpace (5.6.R3) software (18), a literature Visibility App Tool created by Prof. Chaomei Chen for analyzing indicators such as country, journal, institution, author, reference. In addition, CiteSpace was also used to analyze outbreak keywords and visualize them to predict trends in the field. The centrality of a node is a graph-theoretical property that quantifies the importance of the node’s position in a network. A commonly used metric is betweenness centrality (In this study, all centrality refers to betweenness centrality).Betweenness centrality is a concept introduced by the American sociologist Freeman (19),it measures the percentage of shortest paths in the network to which a given node belongs (20). Formula:


$$g\left(\upsilon \right)=\sum_{s\neq \upsilon \neq t}\frac{\sigma_{st}\left(\upsilon \right)}{\sigma_{st}}$$

*g(v)* represents the centrality value of the vertex *v*. *σ_st_* (*v*) represents the number of shortest paths passing through *v* between vertex s and vertex t. *σ_st_* represents the number of all shortest paths from vertex s to vertex t. Burst detection is used to detect significant changes in the number of references at a given time. It is used to discover the decline or rise of a certain subject term or keyword. The algorithm invented by Kleinberg, J was used in cite space to predict the change of keywords at a certain stage and thus to predict the field’s future development (21). The higher the burst strength indicates the higher the frequency of this keyword in the observed time period.

The CiteSpace parameters were set as follows: Time-slicing was chosen from 2012 to 2021, year per slice(1), and all options in the term source were selected, node types were selected one at a time, selection criteria (g-index, g2 ≤ k Si≤gci,k ∈ Z+,k = 25). Each node in the figure indicated an observation including country, institution, author, co-cited literature and keywords. If the publication is issued in cooperation with several countries, institutions and authors, then each country, institution and author is taken into account, which is the basis for the network of institutional, national and author cooperation.

The node size represented the frequency of occurrence; the larger the node, the higher the frequency of occurrence. Connections between nodes represented collaborative, co-occurring or co-referential relationships. The different colors of nodes represented different years; the different colors of circles from inside to outside indicated the years from 2012 to 2021. The outermost purple ring indicated that the node has very high centeredness and was often regarded as a critical point in a specific domain (22). The impact factor (IF) and the 2020 edition of Journal Citation Reports (JCR) were both included in the analysis as crucial indications of the research’s scientific worth (23).

## Results

A total of 1899 publications on CRC immunotherapy were published on the web of science from 2012 to October 31, 2021, including 1454 (76.57%) ARTICLES and 445 REVIEWS (23.43%). The literature covered 66 countries or regions and 367 institutions.

**Figure 2:**
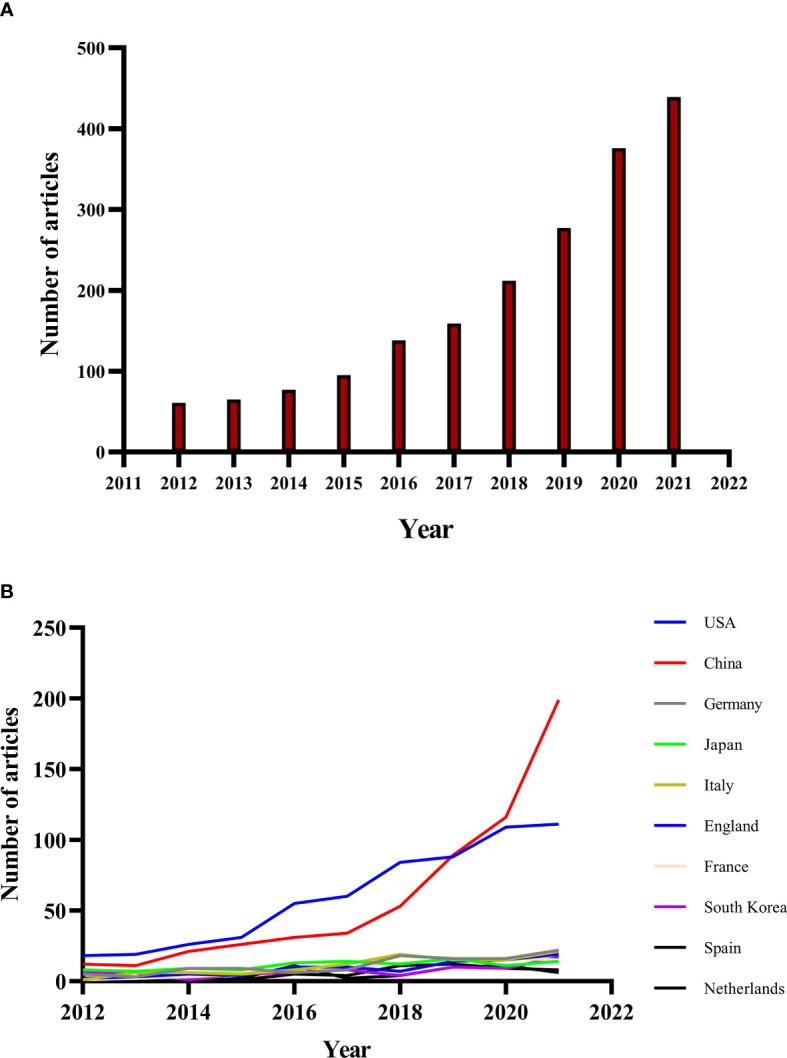
**(A)** Annual publications of immunotherapy in colorectal cancer from 2012 to 2021. **(B)** The country’s annual trend publications related to colorectal cancer immunotherapy from 2012 to 2021.

The annual number of publications for immunotherapy on CRC from 2012 to 2021 as shown in **Figure 2A**, and we divided it into three periods, slow growth period (2012-2015), acceleration period (2016-2017) and rapid growth period (2018-2021). The number of publications increased relatively slowly before 2016 and rapidly after 2016, with more than 150 publications per year and a second acceleration period in 2018. The number of publications on CRC immunotherapy in 2021 was the highest in the last decade, reaching 445 as of October 31, 2021, and the number of publications continued to rise by a large margin.

### Countries and Institutions

**Figure 3:**
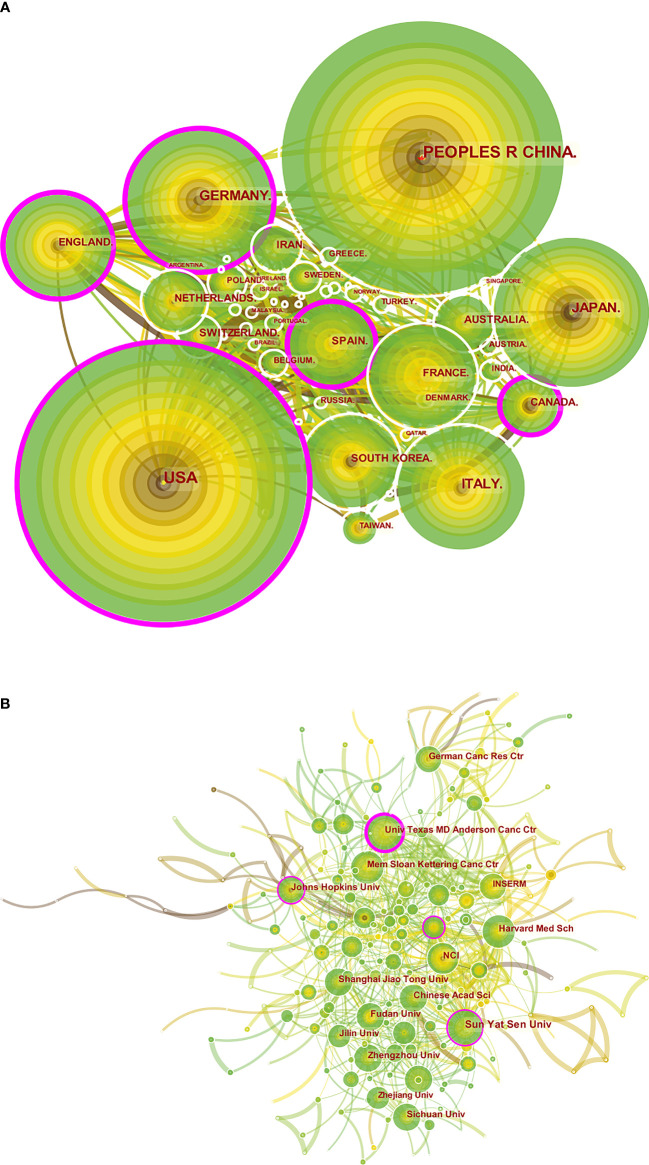
A visual map for CiteSpace network of countries/regions and institutions associated with colorectal cancer immunotherapy. **(A)** Country/regional collaboration analysis; **(B)** Institutional collaboration analysis. The nodes represent countries/regions or institutions, and the lines connect them. Nodes represent countries/regions or institutions. The number of publications grows proportionally to the size of the nodes. The lines between the nodes represent the cooperation relationship, and the thickness of the connecting lines represents the strength of their cooperation, the closer the cooperation, the thicker the connecting lines. the nodes with the outermost purple circles have higher centrality. From 2012 to 2021, the color changes from brown to green.

The annual trend publications associated with immunotherapy for CRC by country from 2012 to 2021 were presented in **Figure 2B**. The United States of America (USA) was the most frequent publisher in this field with 601 publications (31.65 of 1899 publications), followed by China (31.17, with 592 publications), Germany (5.90, with 112 publications), Japan (5.90%, with 112 publications) and Italy (5.69%, with 108 publications) (**Table 1**). The top 5 countries with the highest centrality were: USA, UK, Spain, Canada and Germany (**Figure 3A**). The United States and China contributed 62.82% of the total publications, far more than any other country. China initially had trailed the US in the number of annual publications, but after 2018 its publications in this field have increased rapidly and surpassed the US in 2020, maintaining a high-speed growth rate in 2021. In addition, the annual increase of publications in China was the fastest since 2012, to be followed by the United States, while Japan and the Netherlands grew slowly.

**Table 1:** The top 10 productive countries/regions related to colorectal cancer immunotherapy.

Rank	Country/region	Article counts	Percentage (N/1899)	Citation	Citation per publication
1	United States	601	31.65	19051	31.70
2	China	592	31.17	9285	15.68
3	Germany	112	5.90	3478	31.05
4	Japan	112	5.90	2604	23.25
5	Italy	108	5.69	3671	33.99
6	England	89	4.69	3196	35.91
7	France	86	4.53	4500	52.32
8	South Korea	72	3.79	1168	16.22
9	Spain	56	2.95	1936	34.57
10	Netherlands	49	2.58	1737	33.45

Among the top 10 countries/regions regarding publications, the U.S. had 19,051 citations, far more than all other countries, while it had a high citation/publication ratio (31.65). Although China had many citations (9285), its citation/publication rate (15.68) was lower than other countries. It is to be noted that France had the highest citation/publication ratio (52.32) among the ten countries, despite the relatively low number of publications, which also indicated the high quality of its published papers.

The analysis of the international collaboration network (**Figure 3A**) showed that the United States, which had the highest volume of output, and China worked closely together. China mainly cooperated closely with Singapore and Greece, while the United States largely cooperated with England, France, South Korea, Italy and Spain. China already leads the world in publication output, but its centrality was 0.05, compared to 0.20 in the US.

The top ten activist institutions were listed in **Table 2**. Sun Yat-sen University published 41 papers with 431 citations, followed by the University of Texas M. D. Anderson Cancer Center (33 papers, 1,437 citations) and Fudan University (30 papers, 300 citations). The ten most prolific institutions were from China and the United States, excluding the German Cancer Research Center. Among the top ten productive institutions, the National Cancer Institute (NCI) had the highest citation/publication rate (53.7). Institutional cooperation analysis was produced to reveal the cooperation between institutions (**Figure 3B**).

**Table 2:** Top 10 institutions published literature related to colorectal cancer immunotherapy from 2010 to 2021.

Rank	Institution	Country	Number of studies	Total citations	Average citation
1	Sun Yat-sen University	China	41	431	10.5
2	The University of Texas MD Anderson Cancer Center	USA	33	1437	43.5
3	Fudan University	China	30	424	14.1
4	Shanghai Jiao Tong University	China	30	300	10
5	National Cancer Institute (NCI)	USA	29	1556	53.7
6	Chinese Academy of Sciences	China	29	1113	38.4
7	Harvard Medical School	USA	27	1232	45.6
8	German Cancer Research Center	Germany	27	584	21.6
9	Johns Hopkins University	USA	26	392	15.1
10	Sichuan University	China	26	278	10.7

### Analysis of Journals and Co-Cited Journals

The top ten most prolific and co-cited journals were listed in **Tables 3**, **4**. *Journal for Immunotherapy of Cancer* (66 articles, 3.48%) published the most documents in this field, followed by *Oncoimmunology* (63 articles, 3.32%), *Cancer* (57 articles, 3.00%), *Frontiers in Immunology* (57 articles. 3.00%) and *Cancer Immunology Immunotherapy* (53 articles, 2.79%). Among the top ten most prolific journals, the *Journal for Immunotherapy of Cancer* had the highest IF of 13.751 and *Cancer Research* had the highest citation/publication rate (52.33). Of the top 10 journals, 80% were classified as Q1 (top 25% of IF distribution), remaining two journals were Q2 (between 25th and 50th quartiles) and Q4 (between 50th and 100th quartiles). The most frequently co-cited journal was the Journal of Clinical Oncology (4270 citations), followed most frequently by *Clinical Cancer Research* (3841 citations) and *Cancer Research* (3807 citations). Among the top 10 co-cited journals, *New England Journal of Medicine* was cited 3640 times with the highest IF of 91.253. Except for *Journal of Immunology*, the rest of the journals were in Q1.

**Table 3:** The top 10 productive journals related to colorectal cancer immunotherapy.

Rank	Journal	Article counts	Percentage (N/1899)	Citation per article	IF	Quartile in category
1	Journal For Immunotherapy Of Cancer	66	3.48	14.12	13.751	Q1
2	Oncoimmunology	63	3.32	18.52	8.1	Q1
3	Cancer	57	3.00	8.00	6.86	Q1
4	Frontiers In Immunology	57	3.00	13.91	7.561	Q1
5	Cancer Immunology Immunotherapy	53	2.79	17.62	6.968	Q1
6	Frontiers In Oncology	34	1.79	5.26	6.244	Q2
7	Cancer Immunology Research	31	1.63	32.97	11.151	Q1
8	Clinical Cancer Research	27	1.42	66.11	12.531	Q1
9	Oncology Letters	23	1.21	9.39	2.967	Q4
10	Cancer Research	21	1.11	52.33	12.701	Q1

**Table 4:** The top 10 co-cited journals associated with colorectal cancer immunotherapy.

Rank	Cited Journal	Citation	IF (2020)	Quartile in category
1	Journal of Clinical Oncology	4270	44.544	Q1
2	Clinical Cancer Research	3841	12.531	Q1
3	Cancer Research	3807	12.701	Q1
4	New England Journal of Medicine	3640	91.253	Q1
5	Nature	2792	49.962	Q1
6	Science	2761	47.728	Q1
7	Journal Of Immunology	2445	5.422	Q2
8	Proceedings of the National Academy of Sciences of the United States of America (PNAS)	1867	11.205	Q1
9	Cell	1655	41.584	Q1
10	Nature Medicine	1583	53.44	Q1

**Figure 4:**
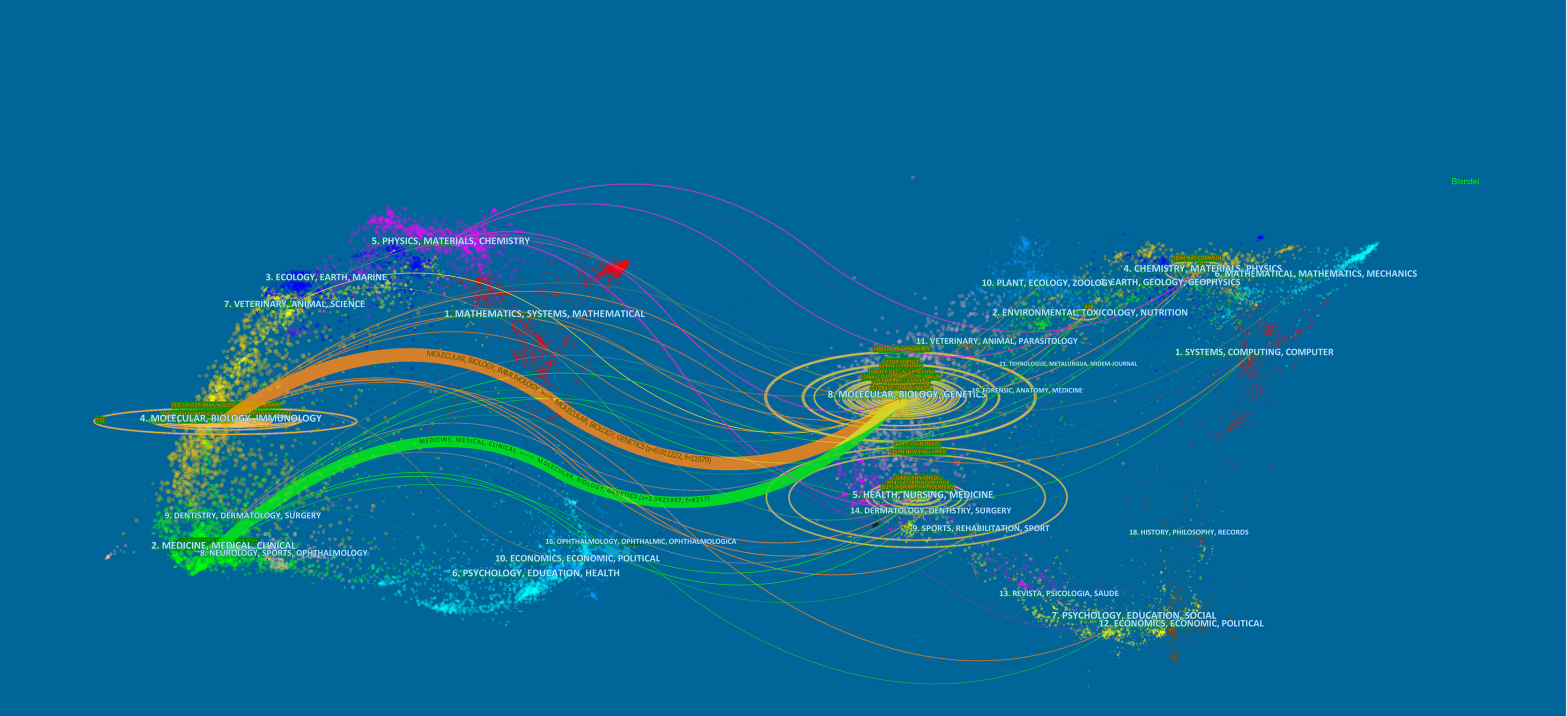
The dual-map overlay of journals related to colorectal cancer immunotherapy. Notes: On the left were the citing journals, on the right were the cited journals, and the colored path represented the citation relationship.

The subject distribution of academic journals was depicted by a dual-map overlay of the journals (24) (**Figure 4**). The citing journal is on the left, and the cited journal is on the right, with the citation relationship indicated by the colored path. The mapping identifies 2 colored primary citation pathways, meaning that researches published to journals in the field of molecular/biology/genetics were primarily cited by researches published in molecular/biology/immunology and medical/medical/clinical journals.

### Analysis of Authors and Co-Cited Authors

**Figure 5:**
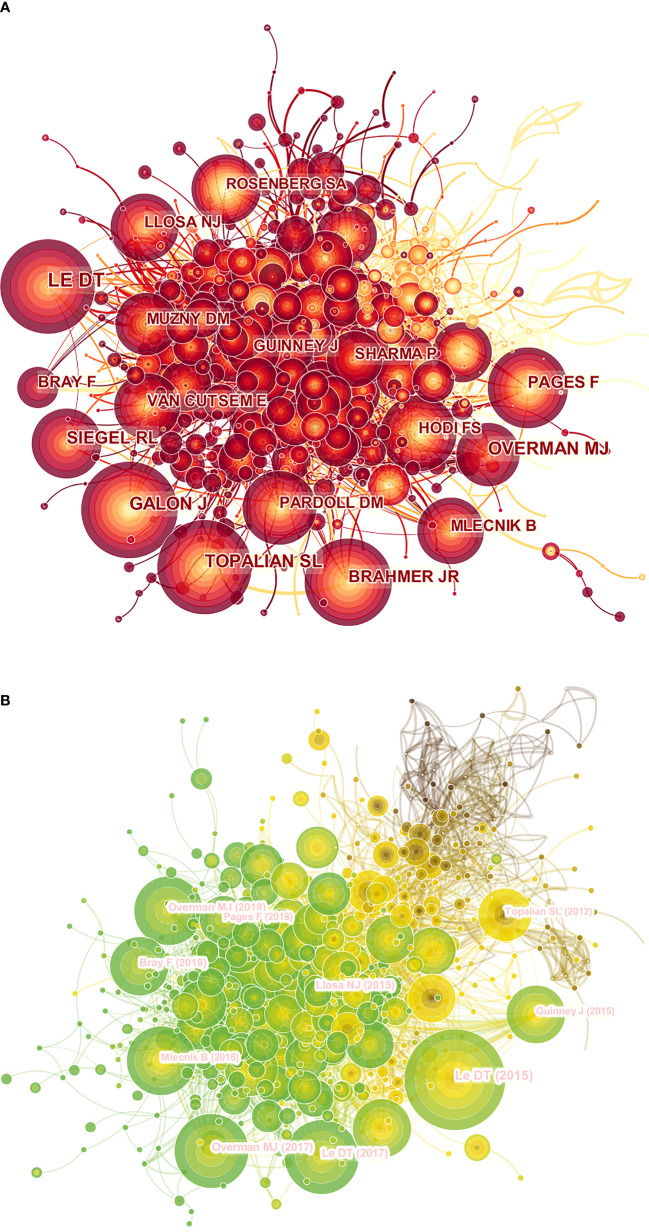
A CiteSpace network visualization of co-cited authors and references regarding colorectal cancer immunotherapy. **(A)** Network visualization diagram of the co-cited authors of the Publications. **(B)** Network visualization diagram of cited references. Co-cited authors or cited references are indicated by the node. The co-citation relationship is indicated by the line connecting the nodes. The node area grows as the number of co-citations increases. The colors represent different years, in A, the color changes from yellow to red from 2012 to 2021, and in B, the color changes from brown to green from 2012 to 2021.

Of all authors who published literature related to colorectal cancer immunotherapy in the last decade, the 10 most productive authors were listed in **Table 5**. Adam E Snook (17 publications) has published the most papers, followed by Scott A Waldman (14 publications) and Michael A Morse (14 publications). Further analysis revealed that six of the top ten authors were from the United States, two were from China, and two were from Germany and France. The co-cited author network was visualized by **Figure 5A**. The most prominent nodes were linked to the most co-cited authors, which including Le DT (567Citation), Topalian SL (314Citation), Galon J (313Citation), Overman MJ (301Citation), and Siegel RL (278Citation) (**Table 5**).

**Table 5:** Top 10 most prolific and co-cited authors in the field of colorectal cancer immunotherapy.

Rank	Author	Count	Location	Rank	Co-cited author	Citation
1	Adam E Snook	17	USA	1	Dung T. Le	567
2	Scott A Waldman	14	USA	2	Topalian SL	314
3	Michael A Morse	14	USA	3	Galon J	313
4	Jefferey Schlom	14	USA	4	Overman MJ	301
5	Kebin Liu	11	China	5	Siegel RL	278
6	Thierry Andre	10	France	6	Brahmer JR	223
7	Niels Halama	10	Germany	7	Pages F	221
8	Dung T Le	10	USA	8	Llosa NJ	198
9	Chunwan Lu	9	USA	9	Bray F	176
10	Yuquan Wei	9	China	10	Mlecnik B	169

### Analysis of Cited References

The co-cited literature network diagram was composed of 850 nodes and 4135 links, with a time slice set to 1 year and a period set to 2012-2021 (**Figure 5B**). According to the five most frequently co-cited articles (**Tables 6**, **7**), PD-1 Blockade in Tumors with Mismatch-Repair Deficiency (with 343 co-citations) published in the *New England Journal of Medicine* (IF=91.253) was the most cited article by Dung T. Le. In addition, 2 of the top 5 co-cited papers were written by Dung T. Le and published in different journals.

**Table 6:** The top five co-cited references related to colorectal cancer immunotherapy.

Rank	Title	Journal IF (2020)	First author	Publication time	Total citations	Quartile in category
1	PD-1 Blockade in Tumors with Mismatch-Repair Deficiency	New England Journal of Medicine (IF=91.253)	Dung T. Le	May,2015	343	Q1
2	Nivolumab in patients with metastatic DNA mismatch repair-deficient or microsatellite instability-high colorectal cancer (Check Mate 142): an open-label, multicentre, phase 2 study	Lancet Oncology (IF=41.316)	Michael J. Overman	July,2017	225	Q1
3	Mismatch-repair deficiency predicts response of solid tumors to PD-1 blockade	Science (IF=47.728)	Dung T. Le	Jun,2017	197	Q1
4	Durable Clinical Benefit with Nivolumab Plus Ipilimumab in DNA Mismatch Repair-Deficient/Microsatellite Instability-High Metastatic Colorectal Cancer	Journal of clinical oncology (IF=44.544)	Michael J Overman	Jan,2018	174	Q1
5	Global cancer statistics 2018: GLOBOCAN estimates of incidence and mortality worldwide for 36 cancers in 185 countries	CA: A Cancer Journal for Clinicians (IF=508.702)	Freddie Bray	September,2018	173	Q1

**Table 7:** The top five centralities of co-cited references related to colorectal cancer immunotherapy.

Rank	Title	Journal IF (2020)	First author	Publication time	Centrality	Quartile in category
1	Nivolumab plus Ipilimumab in Advanced Melanoma	New England Journal Of Medicine (IF=91.253)	Jedd D. Wolchok	Jun,2013	0.19	Q1
2	Characterization of the immunophenotypes and antigenomes of colorectal cancers reveals distinct tumor escape mechanisms and novel targets for immunotherapy	Genome Biology (IF=13.583)	Mihaela Angelova	Mar,2015	0.11	Q
3	Improved survival with ipilimumab in patients with metastatic melanoma.	New England Journal Of Medicine (IF=91.253)	F Stephen Hodi	Aug,2010	0.11	Q1
4	Primary, Adaptive, and Acquired Resistance to Cancer Immunotherapy	Cell (IF=41.584)	Padmanee Sharma	Feb,2017	0.09	Q1
5	Durable Cancer Regression Off-treatment and Effective Re-induction Therapy with an Anti-PD-1 Antibody	Clinical cancer research (IF=12.531)	Evan J. Lipson	Jan,2013	0.09	Q1

**Figure 6:**
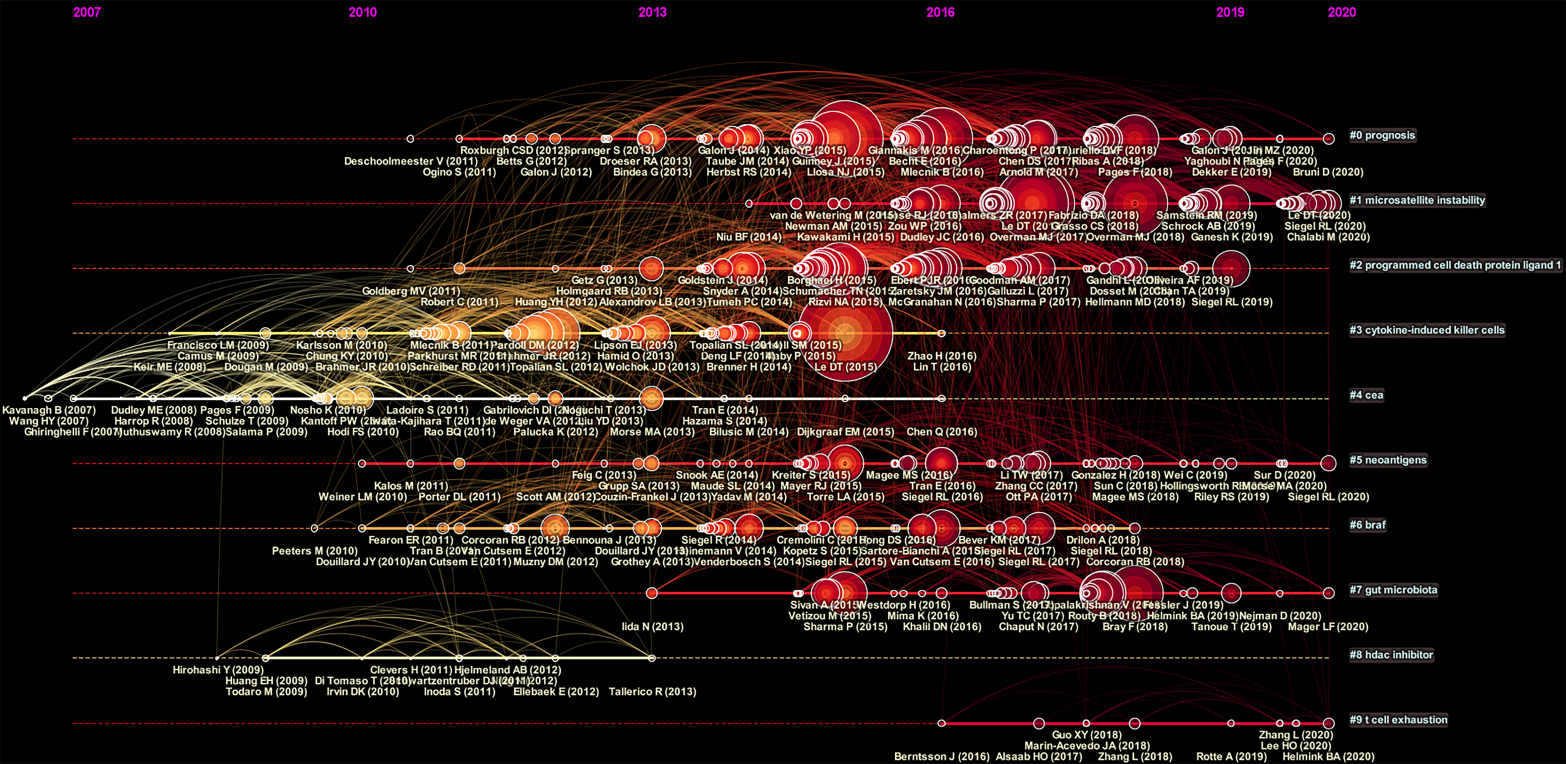
A timeline view for co-cited references associated with colorectal cancer immunotherapy. The node’s position on the horizontal axis represents the time when the reference first appeared, and the node’s size is positively correlated with the number of citations of the reference. The lines between the nodes represent co-cited relationships. The redder the color means closer to 2021, and the more yellow the color means closer to 2012.The clusters with redder colors and larger nodes included more publications, demonstrating that the issue of this cluster was a hot topic in the field.

Moreover, we performed a temporal co-citation analysis and mapped the timeline view of co-cited references (**Figure 6**). We found that “CEA” (Cluster4) was a relatively early hotspot, “cytokine-induced killer cells” (Cluster3) was a mid-term (2008-2016) research hotspot, and “prognosis” (Cluster0) had the darkest color representing the most publications and was a consistent research hotspot for CRC immunotherapy. “Microsatellite instability” (Cluster1), “programmed cell death protein ligand1” (Cluster2), “hormone replacement” (Cluster5), “braf” (Cluster6) and “gut microbiota” (Cluster7) demonstrated that the issues of these clusters are the current new hotspots in the field.

### Analysis of Keyword Co-Occurrence Clustering and Time Zone

**Figure 7:**
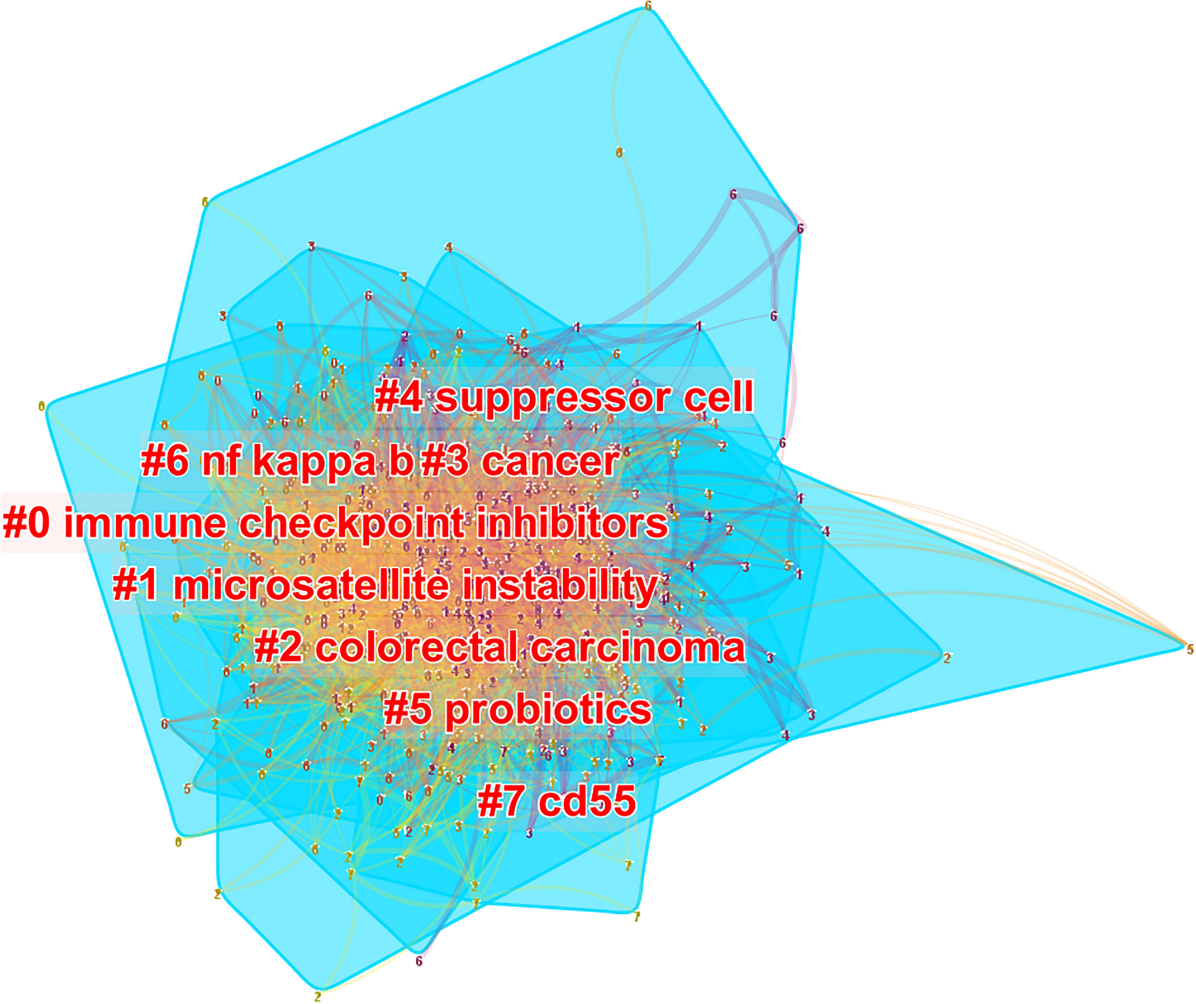
The cluster of keywords related to colorectal cancer immunotherapy. The different colors represented different clusters. Each point represents a keyword, and the number on the node represents the cluster to which the keyword belongs. The lines between the points represent two keywords with co-occurrence relationship. The color of the line segment represents the year, from 2012 to 2021, the color changes from purple to yellow. The blue blocks represent the extent of each cluster in the network space.

Based on the number of citations and centrality analysis of keywords through CiteSpace (**Table 8**), we found that the most popular keywords were Expression, T cell, Dendritic Cell, cell, survival, activation, regulatory t cell, and chemotherapy after removing meaningless keywords (**Table 8**). We also built a network map to visualize keyword clusters (**Figure 7**), and we found that “immune checkpoint inhibitor” (Cluster0), “microsatellite instability” (Cluster1), “suppressor” (Cluster4), “probiotics” (Cluster5), “nf kappa b” (Cluster6) and “cd55” (Cluster7) were the hot spots of research since 2012.

**Table 8:** The top 10 most frequent and centralized keywords related to colorectal cancer immunotherapy.

Rank	Keyword	Counts	Rank	Keyword	Centrality
1	Expression	334	1	Progression	0.12
2	T cell	244	2	Pd 1 blockade	0.10
3	Microsatellite instability	216	3	Mechanism	0.10
4	Dendritic cell	184	4	Combination	0.10
5	Cell	152	5	Chemotherapy	0.09
6	Blockade	139	6	Monoclonal antibody	0.09
7	Survival	135	7	Expression	0.08
8	Activation	120	89	Regulatory t cell	0.08
9	Regulatory t cell	110	9	Receptor	0.08
10	Chemotherapy	109	10	Activation	0.07

**Figure 8:**
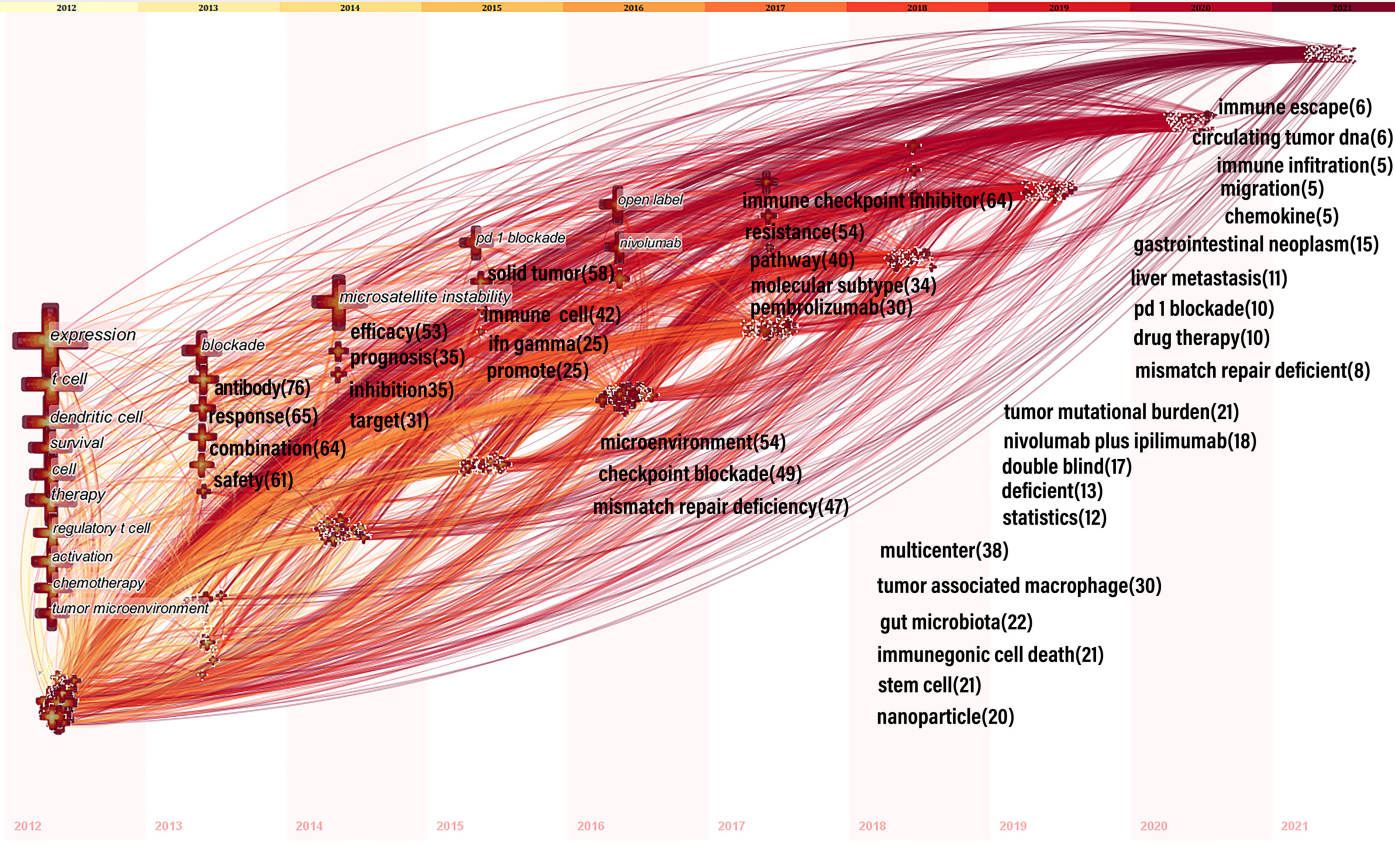
Time zone view of keywords for colorectal cancer immunotherapy. In 2012-2016, keywords with co-occurrence rate ≥80 were shown; from 2013-2021, we manually added annual top5 keywords and their co-occurrence frequency. The position of the cross on the horizontal axis represents the year in which the keyword first appeared. The size of the cross reflects the frequency of co-occurrence, and the larger the cross represents the higher frequency of co-occurrence. The lines between the crosses represent the co-occurrence of two keywords. The lines are colored to indicate the different years. From 2012 to 2021, the color changes from yellow to red.

CiteSpace designed the keyword time zone view to show high-frequency keywords’ evolution clearly. The keywords were situated in the years in which they first appeared together, and colors of the links represented the years when the two keywords first appeared together. High-frequency keywords (T > 80) were displayed in **Figure 8**, as threshold was a cumulative number, resulting in some of the most recent keywords not reaching 80 cumulatively. Therefore, the top five annual high-frequency keywords from 2013 to 2021 were added to complement the time zone view (**Figure 8**).

### Keyword Burst Analysis

**Figure 9:**
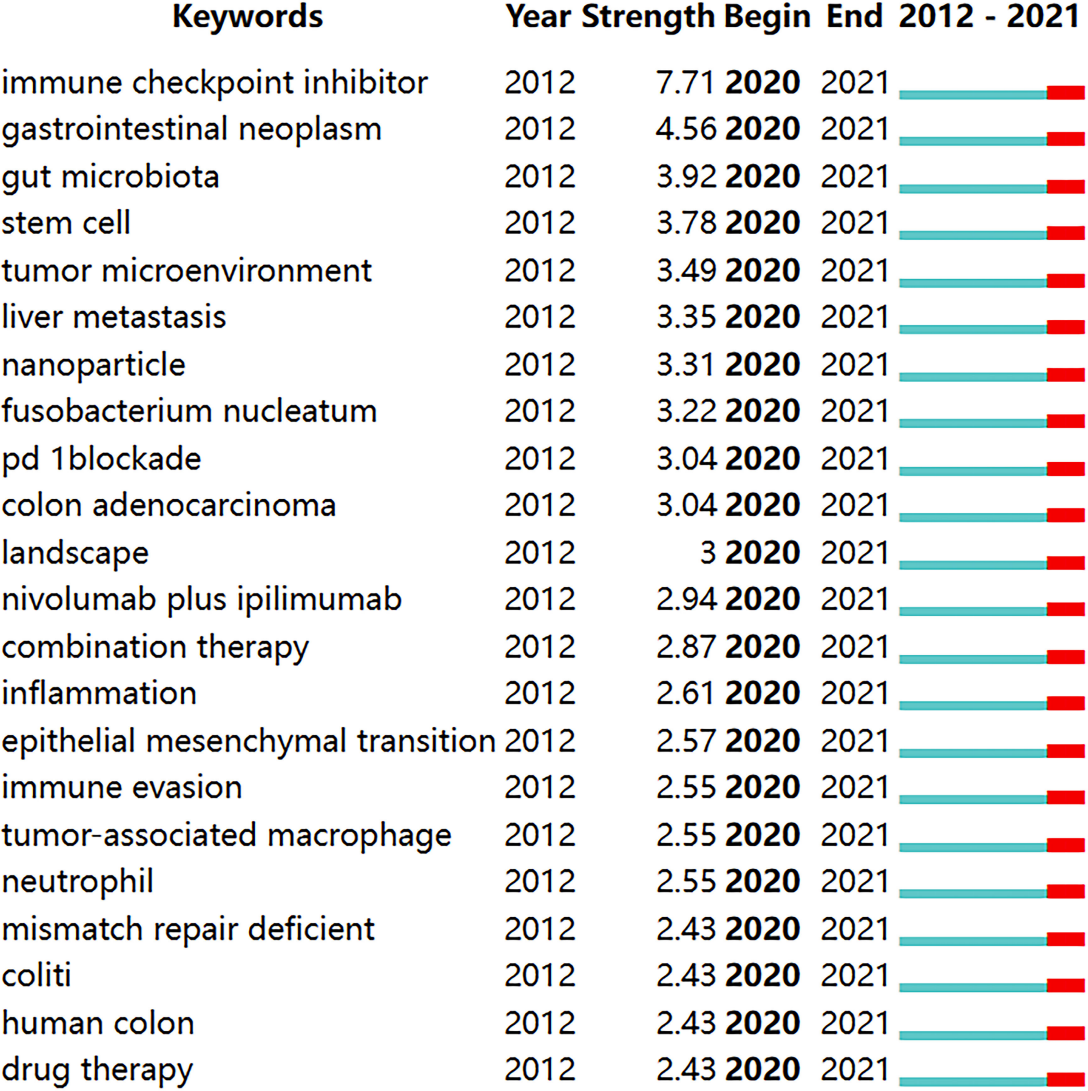
Keywords with burst periods from 2020 onward among the top 103 burst keywords in articles related to colorectal cancer immunotherapy. A blue line indicates the timeline, and the intervals in which bursts were found are indicated by red sections on the blue timeline, indicating the start year, the end year, and the burst duration.

We use CiteSpace to detect emergent keywords to identify the frontiers of research in this field. Among the 103 keywords with high citation outbreaks, we focused on those with bursts starting in 2020 (**Figure 9**), including “immune checkpoint inhibitors” (burst intensity of 7.71), “gut microbiota” (burst intensity of 3. 92), “tumor microenvironment” (burst intensity of 3.49) “liver metastasis” (burst intensity of 3.35), “nanoparticles” (burst intensity of 3.31), “combination therapy” (burst intensity of 2.87).

## Discussion

In this study, we obtained 1899 publications on colorectal cancer immunotherapy from 2012 to 2021 by searching the web of science core database and manual screening. In terms of annual publications, publications increased rapidly after 2015. In terms of national and regional distribution, the United States has been the driving force behind the highest academic contribution to colorectal cancer immunotherapy in the last decade, as reflected by the number of publications, centrality, citation frequency and citation publication ratio. China’s annual publications proliferated after 2016, and since 2020 China has surpassed the United States. Although the average citation was relatively low, the higher total number of citations showed the high contribution of China to the development of the field. France, Spain, Italy and South Korea have lower publication numbers, but their publications have multiplied in recent years, related to the cooperation with the United States.

## Institutions and Institutional Cooperation

90% of the 10 most productive institutions were from China and the United States, indicating that China and the United States played significant roles in the academic development of the field. Interestingly, Sun Yat-sen University, the most productive institution globally, had the lowest average citation among the top ten institutions. The National Cancer Institute (NCI) had the highest total citations (1556) and average citations (53.7) among the top 10 institutions, indicating that NCI published higher quality articles in the field and played a crucial role in promoting the development of the field.

## Journals and Cited Journals

Among the ten most active journals, *Journal for Immunotherapy of Cancer* (66 articles), *Oncoimmunology* (63 articles), *Cancer* (57 articles), *Frontiers in Immunology* (57 articles) and *Cancer Immunology Immunotherapy* (53 articles) published more than 50 papers. The *Journal of Clinical Oncology* received the highest citations. Notably, both *Clinical Cancer Research* (66.11) and *Cancer Research* (52.33) had more than 50 citations per article and ranked 2nd and 3rd, respectively, among co-cited journals. thus, they are recognized as research resources for CRC immunotherapy.

Most of the top 10 productive journals were related to immunology and clinical, while most of the 10 highly co-cited journals were related to biology, which was consistent with the dual-map overlay analysis. The dual-map overlay represented the subject distribution of academic journals. **Figure 4** showed that studies from molecular/biology/genetics co-cited journals were mainly cited by journals published in molecular/biology/immunology and medicine/medical/clinical, which implied that there were two broad main directions of research related to CRC immunotherapy, one direction was focused on basic. The other direction was focused on translational research from basic to clinical. Meanwhile, journals with high IF in the JCR division accounted for the vast majority of the top 10 journals (80%) and co-cited journals (90%) in the first quarter, indicating that these journals were interested in and played an essential role in research related to CRC immunotherapy.

## Authors and Cited Authors

Dung T. Le from Johns Hopkins University was ranked first among all co-cited authors, and his publications in the field of CRC immunotherapy were placed 8th among all authors, indicating his outstanding contribution to the development of the field. Two of his highly cited articles, “PD-1 Blockade in Tumors with Mismatch-Repair Deficiency” and “Mismatch-repair deficiency predicts response of solid tumors to PD-1 blockade”, were published in *New England Journal of Medicine* (IF=91.253) and *Science* (IF=47.728), respectively. Researchers first evaluated the clinical activity of PD-1 inhibitors in patients for progressive CRC with/without mismatch repair (MMR) and found that microsatellite instable (MSI) CRC (MSI-CRC) were more sensitive to PD-1 blockade responses compared to microsatellite stable (MSS) tumors (25). The researchers then expanded the study to evaluate the effects of PD-1 inhibitors in 12 different types of advanced MMR tumors. They found that in mismatch repair deficient(dMMR) tumors, most mutant neoantigens made them sensitive to immune checkpoint blockade, regardless of the origin of the cancer tissue (26). Therefore, these articles were considered reliable resources for future studies on mismatch repair and PD-1.

## Co-cited References

We found that most 5 co-cited articles with the highest frequency and centrality were related to immune checkpoint inhibitors (ICIs) and MSI, which indicated the importance of ICIs and MSI in the field. Among the top five co-cited references, the article titled “Global cancer statistics 2018: GLOBOCAN estimates of incidence and mortality worldwide for 36 cancers in 185 countries” was focused on a global review of cancer prevalence and treatment measures (27). Based on the top 5 cited articles by centrality, there was 1 article analyzed the immunophenotypic and antigenic genomic characterization of CRC, revealing different targets of immune escape and tumor immunity (28). It is well known that immune escape, a process by which tumor cells escape killing by the immune system, was a critical factor affecting the effectiveness of tumor therapy. Therefore, this highly cited paper demonstrated that revealing the mechanism of immune escape may help to discover potential targets for immunotherapy of colon cancer. Another article with a high centrality revealed the mechanism of cancer immunotherapy resistance (29), which indicated that immunotherapy resistance had received much attention in CRC immunotherapy.

The timeline view of the co-cited literature showed that most of the studies were published after 2007. The prognosis (Cluster0) has the darkest color and the most published literature and has been a consistent focus of CRC immunotherapy. Microsatellite instability (Cluster1), programmed cell death protein ligand1 (Cluster2) have appeared in recent years and have larger nodes and warmer colors, indicating that the problems under these clusters were hot research topics in recent research years. Microsatellite instability (Cluster1), programmed cell death protein ligand1 (Cluster2) have emerged in recent years and have larger nodes and warmer colors, indicating that the problems under these clusters were hot spots of research in recent years, which was consistent with the above highly co-cited literature focusing on PD-1/PD-L1 and mismatch repair.In addition, in recent years, cytokine-induced killer cells (Cluster3) and t cell exhaustion (Cluster9) may be new research hotspots in CRC immunotherapy. It has been shown that gut microbes can promote colorectal carcinogenesis by stimulating specific immune responses (30). Gut microbiota (Cluster7) may be a research hotspot in recent years to understand the mechanisms of colorectal carcinogenesis and identify relevant immunotherapeutic strategies. Moreover, hormone replacement (Cluster5) may be one of the hot spots in reducing the risk of colon cancer in MSI type of high-aged menopausal women.

## Keywords

In bibliometric analysis, keyword bursts can reflect research hotspots in academic fields (31), and time zone maps can show the evolution of new hotspots (32). Based on the change in the number of publications, we divided the evolution of keywords into three phases: slow growth period (2012-2014), acceleration period (2015-2017) and rapid development period (2018-2021). During the slow growth period (2012-2014) rising terms mainly covered: “expression”, “t cell”, “dendritic cell”, “regulatory t cell”, “tumor microenvironment”, “blockade”, “antibody”, “microsatellite instability”, “inhibitor”, and “target”, mainly associated with treatments and mechanisms. In the acceleration period (2015-2017), most new terms focus on clinical and mechanistic studies of ICIs, MSI, and drug resistance. These keywords include: “pd 1 blockade”, “ifn gamma”, “open label”, “nivolumab”, “microenvironment”, “checkpoint blockade”, “mismatch repair deficiency”, “immune checkpoint inhibitor”, “resistance”, and “pembrolizumab”. During the rapid development period (2018-2021), emerging topics included “tumor-associated macrophages”, “gut microbiota”, “immunogenic cell death”, “stem cells”, “nanoparticles”, “tumor mutation burden”, “liver metastasis”, “drug therapy”, “immune escape”, and “circulating tumor dna”. These themes inherited the characteristics of the accelerated phase and used novel technologies such as nanomaterials, and gave rise to many research directions such as gut microbes, stem cells, tumor-associated macrophages, and immune escape.

According to the citation count and keyword centrality analysis, which mainly included keywords related to immunotherapy mechanism of CRC included “Microsatellite instability”, “T cell”, “PD-1blockade”. It was similar to Nicolas J. Llosa et al. (33), who reported that in dMMR or microsatellite instability-high (MSI-H) (dMMR/MSI-H) tumors. The major histocompatibility presented on the surface of the tumor cell complex (MHC) class I peptide complex induces the migration of CD8 tumor-infiltrating lymphocytes, helper T cells 1, CD4 T cells and macrophages into the tumor microenvironment to induce IFN-γ secretion for antitumor effects and to balance this active microenvironment, dMMR/MSI-H tumors selected for sustained upregulation of the T cell suppressor ligands PD-L1, CTLA-4, fostering immune escape.

Several studies have confirmed the significant anti-tumor effects of PD-1blockade in dMMR/MSI-H CRC immunotherapy (25, 34), further confirming that these keywords were the current hot topic of research in the field of CRC immunotherapy. A review of CRC immunotherapy by Ahui Fan et al. (35) also focused on the treatment of microsatellite unstable and microsatellite stable CRC, mainly surrounding various therapeutic approaches with ICIs. In addition, most CRC immunotherapies were associated with keywords such as PD-1blockade, MSI, and T cell (2), indicating that these aspects were the hot topics in recent years.

A comprehensive analysis of keyword emergence and timezone view can reveal the hot spots and future research trends in the field of CRC immunotherapy. The keywords that emerged since 2020 include “Immune checkpoint inhibitor”, “pd-1blockade”, “nivolumab plus ipilimumab”, “combination therapy”, and “drug therapy” with burst strength of:7.71, 3.04, 2.94 and 2.43, respectively. These keywords imply that research in these aspects has been prevalent in this field over the past year. Monotherapy of dMMR/MSI-H CRC showed a significant increase in the durable response rate of the drug with a manageable safety profile, but more than 50% of patients were ineffective to treatment (36). Therefore, discovering new strategies to overcome primary ICI resistance was essential to improve its antitumor effect. Combination with other ICIs such as the CTLA-4 inhibitor ipilimumab may enhance the durable clinical benefit of treating dMMR/MSI-H CRC (37). Recent studies have shown that PD-1 inhibitors combined with CTLA-4 prolong OS in advanced refractory CRC (38), which confirmed that combining multiple ICIs may be a hot topic for future research in this field. Unlike dMMR/MSI-H CRC, mismatch repair proficiency(pMMR) or microsatellite instability-low (MSI-L) (pMMR/MSI-L) tumors, which account for 95% of all metastatic colorectal cancer (mCRC) cases, had a lower mutational burden and poorer immune cell recruitment, resulting in a sub-optimal response to ICIs (13). Researchers found that nivolumab combined with ipilimumab observed pathologic responses in 27% of patients with early-stage pMMR tumors (39). Furthermore, since KRAS/NRSA/BRAF wild-type mCRC was associated with increased CTLA-4 and PD-L1 expression for EGFR monoclonal resistance, ICIs combined with EGFR monoclonal antibodies showed good safety and efficacy in the treatment of MSS mCRC (40). Anti-angiogenic drugs can enhance the anti-tumor activity of CD8 T cells by upregulating PD-L1 expression and reducing immunosuppressive cells such as TAM and Treg. Several studies have shown encouraging ICIs in combination with the anti-angiogenic agent regorafenib in pMMR-MSS (41). Radiation-induced cell injury may increase response to immunotherapy through *in vitro* effects. In a study by Aparna Raj Parikh et al. (42), CTLA-4 and PD-1 dual blockade combined with radiotherapy showed encouraging efficacy in treating patients with pMMR/MSI-L mCRC. In addition, several trials of ICIs in combination with bispecific antibody and MEK were in progress (NCT03428126, NCT03271047, NCT04137289). These studies suggested that combining other therapeutic modalities such as chemotherapy, antigenic combinations, MEK, bispecific antibody, and radiotherapy may increase the immunotherapy response. It was similar to the results reported by Wang et al. (35)and further confirmed that ICIs in combination with other therapeutic modalities were the most popular direction of research in this field. Keyword burst and clustering showed that “immune checkpoint inhibitors”, “combination therapy”, and “drug therapy” were hot topics in this field and promised to be studied more deeply in the future.

The liver was the most common location of distant metastases from mCRC and the central organ of immune tolerance (43, 44). In a preclinical model of liver metastasis from CRC, liver metastases gathered activated CD8 T cells from the body circulation and liver myeloid cells induced T cell apoptosis *via* the Fas-FasL signaling pathway, resulting in the systemic immune desert (45). The results of another study showed that liver metastasis induced tumor-specific immunosuppression in distant tumors by activating Tregs and regulating CD11b monocytes in a preclinical model of CRC (46). Fujiyoshi et al. found that MSS-mCRC patients were more likely to present with liver metastases than MSI-H mCRC patients (71.0% vs 26.7%, p=0.001) (47). Thus, liver metastases may be one of the reasons for the ineffectiveness of ICI therapy in patients with MSS mCRC, which was supported by a recent study by Want et al. (48). Therefore, an in-depth investigation into the mechanisms of drug resistance development in CRC liver metastasis models may focus on future research.

The tumor microenvironment was the internal environment for tumor cell growth and metastasis, mainly composed of endothelial cells, immune cells, and fibroblasts (49). There was accumulating evidence that many suppressor cells in the tumor microenvironment hindered tumor killing by immune cells and caused the immune escape of tumor cells. Tumor-associated macrophages (TAM) were innate immune cells, accounting for 50% of the human tumor cell mass, and were divided into tumor suppressor M1 and tumor promoter M2. It has been shown that M2 was strongly associated with epithelial-mesenchymal transition and chemoresistance in CRC (50). In addition, TAM suppressed T-cell responses and thus diminished the effectiveness of ICIs (51). Targeting TAM seemed to be a promising treatment modality. Neutrophil extracellular traps (NETs) were reticulated structures consisting of chromatin DNA, dispersed cytoplasm and granular proteins squeezed by activated neutrophils to capture and kill bacteria (52). Zhang et al. (53) reported that inhibition of NETs in a mouse model increased CD8+ T cell infiltration and toxicity, thereby overcoming PD-1 resistance. Comito et al. (54, 55) showed that tumor cells modified the microenvironment by reducing tumor-specific T cells and converting some killer T cells into regulatory T cells (Tregs),which expressed high levels of inhibitory receptors (e.g. PD-1), thus generating an immunosuppressive microenvironment hindering immune killing. In addition, suppressor cells in the tumor microenvironment, such as myeloid-derived suppressor cells and tumor-associated stromal cells, are also resistant to anti-tumor T-cell responses to sustain tumor immune tolerance (56, 57). Therefore, it is critical to explore the CRC tumor microenvironment and reshape the immune microenvironment to improve tumor-infiltrating T cells in CRC immunotherapy. Keyword clustering and bursting showed that topics such as “tumor microenvironment”, “neutrophil”, “tumor-associated macrophage”, “suppressor cell”, and “immune evasion” were considered as new research hotspots.

As technology advanced, many technologies drove the development of immunotherapy for CRC. Nanotechnology-based antioxidants and therapeutics were considered the next generation of cancer treatment tools (22). Duan et al. (58) synthesized OxPt/DHA nanoparticles, which significantly enhanced the efficacy of ICIs by increasing the uptake of DHA and OxPt precursors by CRC cells and enhancing the infiltration of CD8+ T cells within the tumor. In addition, Xu et al. (59) used converted nanoparticles containing the photosensitizer chlorine e6 and Toll-like-receptor-7 agonist combined with an immune checkpoint inhibitor that generated potent anti-tumor immunity in CRC cells and exhibited long-term immune memory capacity. The review by Oliveira et al. showed that nanomaterials have emerged as a new direction for CRC immunotherapy. With the development of sequencing technology, researchers have found that tumor mutational burden was an independent predictor of the treatment efficacy with ICIs. In clinical trials of CRC treated by PD-L1 combined with CTLA-4, higher TMB was associated with better OS (38). Therefore, researches related to nanomaterials and tumor mutation burden were expected to be further explored in the future.

An increasing number of studies have shown that gut microbes play essential roles in cancer development. Tanoue et (60) showed that anti-PD-1 treatment was significantly improved after inoculation of a consortium of 11 bacteria in a mouse model of MC38 colon adenocarcinoma. In addition, Marge et al. (61) used a Bifidobacterium pseudolongum combined with an ICIs treatment regimen in germ-free mice bearing colon cancer cells, which increased T-cell activation and reduced tumor volume in the animals. These studies showed a promising future of gut microbes in CRC immunotherapy. Recently, significant leaps have been made to study gut microbes in overcoming ICB acquired drug resistance. Baruch et al. (62) showed that FMT from CR donors combined with anti-PD-1 therapy-induced clinical responses in patients with anti- PD-1 refractory metastatic melanoma. Davar et al. (63) found that 40% of melanoma patients could overcome acquired resistance to anti-PD-1 therapy with FMT treatment. All of these data indicated the critical role of gut microbes in immunotherapy. Keyword bursts indicated that “gut microbiota” would be a new research hotspot in the field of CRC immunotherapy, and in the future, more and more attention would be focused on gut microbiota.

Taking our research and discussions together, we believe there are some aspects of clinical work that should be noted. Defective DNA mismatch repair (MMR) can be detected either by the lack of immunohistochemical staining of the MMR proteins MLH1, MSH2, MSH6 or PMS2 or by PCR-identified alterations in the lengths of microsatellites between a patient’s tumour and a sample of normal tissue or blood. The use of immunotherapy for dMMR/MSI-H CRC may lead to better outcomes, and the combination of multiple immune checkpoint inhibitors may achieve satisfactory results compared to single agents. Patients should be observed for immune-related side effects during treatment, such as immune-related nephritis, immune-related endocrine disorders and toxicity. However, there are still some challenges in the clinical treatment of this population, such as the keyword “resistance” that appeared in **Figure 8** 2018, suggesting that some colorectal cancer patients in the clinic become resistant to immunotherapy drugs. There is still no good way to overcome drug resistance and recover the effectiveness of immunotherapy. For patients with pMMR-MSI-L colorectal cancer, combination therapy with PD1 inhibitors and other immune checkpoint molecular modulators (e.g., CTLA4) may benefit a small subset of patients with pMMR-MSI-L tumors, but for most patients with this CRC subtype, an alternative approach to immunomodulation is necessary. For example, the combination of Bispecific antibody therapy, radiation therapy, anti-angiogenic drugs. Overall, finding ways to increase the immune cells infiltrating the tumor environment is critical to their immunotherapy for this category of patients. In addition, tumor mutational burden is an independent factor in predicting the efficacy of ICI treatment. In clinical trials of treating CRC with PD-L1 in combination with CTLA-4, higher TMB was associated with better OS, so stratification of patients by immune mutational load prior to treatment may help determine patient treatment efficacy and thus contribute to the selection of therapeutic agents.

## Limitations

We should explain the limitations of the study. On the one hand, these data are collected from a single source, the web of science core, only, leading to the omission of articles from other sources. In addition, the manual removal of papers unrelated to the study by the investigator may lead to selection bias. Last, only English articles were entered in this study, which may lead to source bias.

## Conclusion

To our knowledge, our study was the first comprehensive metrological and statistical analysis of CRC immunotherapy over the past 10 years. In this study, we found that the United States has contributed significantly to the advancement of the field by publishing the most significant number of articles in the field and keeping the quality of articles high. China’s publications in this field snowballed after 2018, surpassing the U.S. since 2020, and maintaining a rapid growth trend that may be related to government financial support. The rapid growth of publications also indicated that CRC immunotherapy was becoming of increasing interest worldwide.

Our research showed a trend for cooperation between various countries and institutions. However, there was a still lack of intensive cooperation between different institutions/countries. Notably, the National Cancer Institute (NCI) and Sun Yat-sen University have made significant contributions to the progress of the field. Therefore, institutions can strengthen their cooperation with them to promote the future development of the field better.

*Journal of Clinical Oncology* and *Journal for Immunotherapy of Cancer* were the most cited and most published journals. In addition, the most prolific and co-cited authors were Adam E Snook and Dung T. Le, respectively. Thus, researchers can better grasp the research progress in this field through articles published in these journals or authors who will be potential collaborators in the field.

Our research indicated that nanomaterials might be the most advanced and popular technology in recent research. In addition, CRC immunotherapy is associated with microsatellite status. Research on immunotherapy for MSI-CRC has focused on PD-1blockade, T cell and combination with other ICIs. Research on immunotherapy for microsatellite stabilized CRC has focused on immune checkpoint inhibitors, combination therapy, and drug therapy. These aspects will remain significant research hotspots in the future. Colorectal cancer liver metastasis has received increasing attention from researchers due to its characteristics. In addition, research on the immune microenvironment has drawn attention to its vital role in immunotherapy, and improving the effectiveness of immunotherapy by modulating the immune microcircuit has become increasingly important in the treatment of CRC. We can predict that the research on tumor microenvironment will become more enthusiastic in the future. The role of gut microbes in CRC immunotherapy may soon become a research hotspot and should be closely monitored.

## Data Availability Statement

The datasets presented in this study can be found in online repositories. The names of the repository/repositories and accession number(s) can be found in the article/supplementary material.

## Author Contributions

LM: Writing- Original draft preparation, Investigation, table and figure preparation. JM: Investigation and table preparation. MT : Supervision. YL: Conceptualization, Methodology, Supervision. All authors contributed to the article and approved the submitted version.

## Funding

This work was supported by National Natural Science Foundation of China (31770537) and Major Science and Technology Special Project of Gansu Province (20ZD7FA003).

## Conflict of Interest

The authors declare that the research was conducted in the absence of any commercial or financial relationships that could be construed as a potential conflict of interest.

## Publisher’s Note

All claims expressed in this article are solely those of the authors and do not necessarily represent those of their affiliated organizations, or those of the publisher, the editors and the reviewers. Any product that may be evaluated in this article, or claim that may be made by its manufacturer, is not guaranteed or endorsed by the publisher.

## References

[1] Sung H Ferlay J Siegel RL Laversanne M Soerjomataram I Jemal A. Global Cancer Statistics 2020: GLOBOCAN Estimates of Incidence and Mortality Worldwide for 36 Cancers in 185 Countries. CA Cancer J Clin (2021) 71(3):209–49. doi: 10.3322/caac.21660 33538338

[2] Ganesh K Stadler ZK Cercek A Mendelsohn RB Shia J Segal NH. Immunotherapy in Colorectal Cancer: Rationale, Challenges and Potential. Nat Rev Gastroenterol Hepatol (2019) 16(6):361–75. doi: 10.1038/s41575-019-0126-x PMC7295073 30886395

[3] Sveen A Kopetz S Lothe RA. Biomarker-Guided Therapy for Colorectal Cancer: Strength in Complexity. Nat Rev Clin Oncol (2020) 17(1):11–32. doi: 10.1038/s41571-019-0241-1 31289352 PMC7577509

[4] Giordano G Remo A Porras A Pancione M. Immune Resistance and EGFR Antagonists in Colorectal Cancer. Cancers (Basel). (2019) 11(8):1089. doi: 10.3390/cancers11081089 PMC6721348 31370270

[5] Hinshaw DC Shevde LA. The Tumor Microenvironment Innately Modulates Cancer Progression. Cancer Res (2019) 79(18):4557–66. doi: 10.1158/0008-5472.CAN-18-3962 PMC6744958 31350295

[6] Chmielik E. Pathology and Tumor Microenvironment: Past, Present, and Future. Pathobiology (2020) 87(2):55–7. doi: 10.1159/000507222 32289800

[7] Zhang H Chen J. Current Status and Future Directions of Cancer Immunotherapy. J Cancer (2018) 9(10):1773–81. doi: 10.7150/jca.24577 PMC5968765 29805703

[8] Liu J Chen Z Li Y Zhao W Wu J Zhang Z. PD-1/PD-L1 Checkpoint Inhibitors in Tumor Immunotherapy. Front Pharmacol (2021) 12:731798. doi: 10.3389/fphar.2021.731798 34539412 PMC8440961

[9] Alsaab HO Sau S Alzhrani R Tatiparti K Bhise K Kashaw SK. PD-1 and PD-L1 Checkpoint Signaling Inhibition for Cancer Immunotherapy: Mechanism, Combinations, and Clinical Outcome. Front Pharmacol (2017) 8:561. doi: 10.3389/fphar.2017.00561 28878676 PMC5572324

[10] Zhang H Dai Z Wu W Wang Z Zhang N Zhang L. Regulatory Mechanisms of Immune Checkpoints PD-L1 and CTLA-4 in Cancer. J Exp Clin Cancer Res (2021) 40:184. doi: 10.1186/s13046-021-01987-7 34088360 PMC8178863

[11] Agarwal P Le DT Boland PM. Immunotherapy in Colorectal Cancer. Adv Cancer Res (2021) 151:137–96. doi: 10.1016/bs.acr.2021.03.002 34148613

[12] Yang Y. Cancer Immunotherapy: Harnessing the Immune System to Battle Cancer. J Clin Invest. (2015) 125:3335–7. doi: 10.1172/JCI83871 PMC4588312 26325031

[13] Kim CW Chon HJ Kim C. Combination Immunotherapies to Overcome Intrinsic Resistance to Checkpoint Blockade in Microsatellite Stable Colorectal Cancer. Cancers (Basel). (2021) 13(19):4906. doi: 10.3390/cancers13194906 34638390 PMC8507875

[14] Ahn SK Hwang JW. Global Trends in Immunotherapy Research on Breast Cancer Over the Past 10 Years. J Oncol (2020) 2020:4708394. doi: 10.1155/2020/4708394 33204263 PMC7661143

[15] Wang S Wu K Zhang Z Xu Z Wu J Xu S. Mapping Theme Trends and Recognizing Research Hot Spots in the Use of Ultrasound in Orthopaedics: A Bibliometric Analysis of Global Research. Am J Transl Res (2021) 13(8):9892–911. PMC8430154 34540126

[16] Ke L Lu C Shen R Lu T Ma B Hua Y. Knowledge Mapping of Drug-Induced Liver Injury: A Scientometric Investigation (2010-2019). Front Pharmacol (2020) 11:842. doi: 10.3389/fphar.2020.00842 32581801 PMC7291871

[17] Aggarwal A Lewison G Idir S Peters M Aldige C Boerckel W. The State of Lung Cancer Research: A Global Analysis. J Thorac Oncol (2016) 11(7):1040–50. doi: 10.1016/j.jtho.2016.03.010 27013405

[18] Chen C Song M. Visualizing a Field of Research: A Methodology of Systematic Scientometric Reviews. PloS One (2019) 14(10):e0223994. doi: 10.1371/journal.pone.0223994 31671124 PMC6822756

[19] Brandes U. A Faster Algorithm for Betweeness Centrality. J Math Sociol (2001) 25:163–77. doi: 10.1080/0022250x.2001.9990249

[20] Chen C. CiteSpace II: Detecting and Visualizing Emerging Trends and Transient Patterns in Scientific Literature. J Am Soc Infor Sci Technol (2006) 57:359–77. doi: 10.1002/asi.20317

[21] Kleinberg J. Bursty and Hierarchical Structure in Streams *. Data Min Knowl Discov (2002) 7(4):91–101. doi: 10.1145/775060.775061

[22] Chen H Li R Zhang F Yao Q Guo Y. A Scientometric Visualization Analysis for Natural Products on Cancer Research From 2008 to 2020. Front Pharmacol (2021) 12:650141. doi: 10.3389/fphar.2021.650141 34421584 PMC8377543

[23] Li T Yang A Liu G Zou S Chen Y Ni B. Status Quo and Research Trends of Craniopharyngioma Research: A 10-Year Bibliometric Analyses (From 2011 to 2020). Front Oncol (2021) 11:744308. doi: 10.3389/fonc.2021.744308 34660308 PMC8516404

[24] Zhang J Song L Xu L Fan Y Wang T Tian W. Knowledge Domain and Emerging Trends in Ferroptosis Research: A Bibliometric and Knowledge-Map Analysis. Front Oncol (2021) 11:686726. doi: 10.3389/fonc.2021.686726 34150654 PMC8209495

[25] Le DT Uram JN Wang H Bartlett BR Kemberling H Eyring AD. PD-1 Blockade in Tumors With Mismatch-Repair Deficiency. N Engl J Med (2015) 372(26):2509–20. 10.1056/NEJMoa1500596 PMC4481136 26028255

[26] Le DT Durham JN Smith KN Wang H Bartlett BR Aulakh LK. Mismatch Repair Deficiency Predicts Response of Solid Tumors to PD-1 Blockade. Science (2017) 357(6349):409–13. doi: 10.1126/science.aan6733 PMC5576142 28596308

[27] Bray F Ferlay J Soerjomataram I Siegel RL Torre LA Jemal A. Global Cancer Statistics 2018: GLOBOCAN Estimates of Incidence and Mortality Worldwide for 36 Cancers in 185 Countries. CA Cancer J Clin (2018) 68(6):394–424. doi: 10.3322/caac.21492 30207593

[28] Angelova M Charoentong P Hackl H Fischer ML Snajder R Krogsdam AM. Characterization of the Immunophenotypes and Antigenomes of Colorectal Cancers Reveals Distinct Tumor Escape Mechanisms and Novel Targets for Immunotherapy. Genome Biol (2015) 16(1):64. doi: 10.1186/s13059-015-0620-6 25853550 PMC4377852

[29] Sharma P Hu-Lieskovan S Wargo JA Ribas A. Primary, Adaptive, and Acquired Resistance to Cancer Immunotherapy. Cell (2017) 168(4):707–23. doi: 10.1016/j.cell.2017.01.017 PMC5391692 28187290

[30] Yang Y Li L Xu C Wang Y Wang Z Chen M. Cross-Talk Between the Gut Microbiota and Monocyte-Like Macrophages Mediates an Inflammatory Response to Promote Colitis-Associated Tumourigenesis. Gut (2020) 70(8):1495–506. doi: 10.1136/gutjnl-2020-320777 PMC8292576 33122176

[31] Xiao F Li C Sun J Zhang L. Knowledge Domain and Emerging Trends in Organic Photovoltaic Technology: A Scientometric Review Based on CiteSpace Analysis. Front Chem (2017) 5:67. doi: 10.3389/fchem.2017.00067 28966923 PMC5605557

[32] Liu G Jiang R Jin Y. Sciatic Nerve Injury Repair: A Visualized Analysis of Research Fronts and Development Trends. Neural Regener Res (2014) 9(18):1716–22. doi: 10.4103/1673-5374.141810 PMC4211194 25374595

[33] Llosa NJ Cruise M Tam A Wicks EC Hechenbleikner EM Taube JM. The Vigorous Immune Microenvironment of Microsatellite Instable Colon Cancer is Balanced by Multiple Counter-Inhibitory Checkpoints. Cancer Discovery (2015) 5(1):43–51. doi: 10.1158/2159-8290.CD-14-0863 25358689 PMC4293246

[34] André T Shiu KK Kim TW Jensen BV Jensen LH Punt C. Pembrolizumab in Microsatellite-Instability-High Advanced Colorectal Cancer. N Engl J Med (2020) 383(23):2207–18. doi: 10.1056/NEJMoa2017699 33264544

[35] Fan A Wang B Wang X Nie Y Fan D Zhao X. Immunotherapy in Colorectal Cancer: Current Achievements and Future Perspective. Int J Biol Sci (2021) 17(14):3837–49. doi: 10.7150/ijbs.64077 PMC8495390 34671202

[36] Rotte A. Combination of CTLA-4 and PD-1 Blockers for Treatment of Cancer. J Exp Clin Cancer Res (2019) 38(1):255. doi: 10.1186/s13046-019-1259-z 31196207 PMC6567914

[37] Overman MJ Lonardi S Wong K Lenz HJ Gelsomino F Aglietta M. Durable Clinical Benefit With Nivolumab Plus Ipilimumab in DNA Mismatch Repair-Deficient/Microsatellite Instability-High Metastatic Colorectal Cancer. J Clin Oncol (2018) 36(8):773–9. doi: 10.1200/JCO.2017.76.9901 29355075

[38] Chen EX Jonker DJ Loree JM Kennecke HF Berry SR Couture F. Effect of Combined Immune Checkpoint Inhibition vs Best Supportive Care Alone in Patients With Advanced Colorectal Cancer: The Canadian Cancer Trials Group CO.26 Study. JAMA Oncol (2020) 6(6):831–8. doi: 10.1001/jamaoncol.2020.0910 PMC7206536 32379280

[39] Chalabi M Fanchi LF Dijkstra KK Van den Berg JG Aalbers AG Sikorska K. Neoadjuvant Immunotherapy Leads to Pathological Responses in MMR-Proficient and MMR-Deficient Early-Stage Colon Cancers. Nat Med (2020) 26(4):566–76. doi: 10.1038/s41591-020-0805-8 32251400

[40] Lee MS Loehrer PJ Imanirad I Cohen S Ciombor KK Moore DT. Phase II Study of Ipilimumab, Nivolumab, and Panitumumab in Patients With KRAS/NRAS/BRAF Wild-Type (WT) Microsatellite Stable (MSS) Metastatic Colorectal Cancer (mCRC). J Clin Oncol (2021) 39(3):7. doi: 10.1200/JCO.2021.39.3_suppl.7 33275489

[41] Fukuoka S Hara H Takahashi N Kojima T Kawazoe A Asayama M. Regorafenib Plus Nivolumab in Patients With Advanced Gastric or Colorectal Cancer: An Open-Label, Dose-Escalation, and Dose-Expansion Phase Ib Trial (REGONIVO, Epoc1603). J Clin Oncol (2020) 38(18):2053–61. doi: 10.1200/JCO.19.03296 32343640

[42] Parikh AR Clark JW Wo J Yeap BY Allen JN Blaszkowsky LS. A Phase II Study of Ipilimumab and Nivolumab With Radiation in Microsatellite Stable (MSS) Metastatic Colorectal Adenocarcinoma (mCRC). J Clin Oncol (2019) 37(15):3514. doi: 10.1200/JCO.2019.37.15_suppl.3514

[43] Kim CG Kim C Yoon SE Kim KH Choi SJ Kang B. Hyperprogressive Disease During PD-1 Blockade in Patients With Advanced Hepatocellular Carcinoma. J Hepatol (2021) 74(2):350–9. doi: 10.1016/j.jhep.2020.08.010 32810553

[44] Hegde PS Chen DS. Top 10 Challenges in Cancer Immunotherapy. Immunity (2020) 52(1):17–35. doi: 10.1016/j.immuni.2019.12.011 31940268

[45] Yu J Green MD Li S Sun Y Journey SN Choi JE. Liver Metastasis Restrains Immunotherapy Efficacy *via* Macrophage-Mediated T Cell Elimination. Nat Med (2021) 27(1):152–64. doi: 10.1038/s41591-020-1131-x PMC8095049 33398162

[46] Lee JC Mehdizadeh S Smith J Young A Mufazalov IA Mowery CT. Regulatory T Cell Control of Systemic Immunity and Immunotherapy Response in Liver Metastasis. Sci Immunol (2020) 5(52):eaba0759. doi: 10.1126/sciimmunol.aba0759 33008914 PMC7755924

[47] Fujiyoshi K Yamamoto G Takenoya T Takahashi A Arai Y Yamada M. Metastatic Pattern of Stage IV Colorectal Cancer With High-Frequency Microsatellite Instability as a Prognostic Factor. Anticancer Res (2017) 37(1):239–47. doi: 10.21873/anticanres.11313 28011498

[48] Wang C Sandhu J Ouyang C Ye J Lee PP Fakih M. Clinical Response to Immunotherapy Targeting Programmed Cell Death Receptor 1/Programmed Cell Death Ligand 1 in Patients With Treatment-Resistant Microsatellite Stable Colorectal Cancer With and Without Liver Metastases. JAMA Netw Open (2021) 4(8):e2118416. doi: 10.1001/jamanetworkopen.2021.18416 34369992 PMC8353537

[49] Xia A Zhang Y Xu J Yin T Lu XJ. T Cell Dysfunction in Cancer Immunity and Immunotherapy. Front Immunol (2019) 10:1719. doi: 10.3389/fimmu.2019.01719 31379886 PMC6659036

[50] Zhang X Chen Y Hao L Hou A Chen X Li Y. Macrophages Induce Resistance to 5-Fluorouracil Chemotherapy in Colorectal Cancer Through the Release of Putrescine. Cancer Lett (2016) 381(2):305–13. doi: 10.1016/j.canlet.2016.08.004 27514455

[51] Li X Liu R Su X Pan Y Han X Shao C. Harnessing Tumor-Associated Macrophages as Aids for Cancer Immunotherapy. Mol Cancer (2019) 18(1):177. doi: 10.1186/s12943-019-1102-3 31805946 PMC6894344

[52] Ravindran M Khan MA Palaniyar N. Neutrophil Extracellular Trap Formation: Physiology, Pathology, and Pharmacology. Biomolecules (2019) 9(8):365. doi: 10.3390/biom9080365 PMC6722781 31416173

[53] Zhang H Wang Y Onuma A He J Wang H Xia Y. Neutrophils Extracellular Traps Inhibition Improves PD-1 Blockade Immunotherapy in Colorectal Cancer. Cancers (Basel) (2021) 13(21). doi: 10.3390/cancers13215333 PMC8582562 34771497

[54] Lim WC Olding M Healy E Millar TM. Human Endothelial Cells Modulate CD4(+) T Cell Populations and Enhance Regulatory T Cell Suppressive Capacity. Front Immunol (2018) 9:565. doi: 10.3389/fimmu.2018.00565 29628925 PMC5876242

[55] Comito G Iscaro A Bacci M Morandi A Ippolito L Parri M. Lactate Modulates CD4(+) T-Cell Polarization and Induces an Immunosuppressive Environment, Which Sustains Prostate Carcinoma Progression *via* TLR8/miR21 Axis. Oncogene (2019) 38(19):3681–95. doi: 10.1038/s41388-019-0688-7 30664688

[56] Yin K Xia X Rui K Wang T Wang S. Myeloid-Derived Suppressor Cells: A New and Pivotal Player in Colorectal Cancer Progression. Front Oncol (2020) 10:610104. doi: 10.3389/fonc.2020.610104 33384962 PMC7770157

[57] Jiang P Gu S Pan D Fu J Sahu A Hu X. Signatures of T Cell Dysfunction and Exclusion Predict Cancer Immunotherapy Response. Nat Med (2018) 24(10):1550–8. doi: 10.1038/s41591-018-0136-1 PMC6487502 30127393

[58] Duan X Chan C Han W Guo N Weichselbaum RR Lin W. Immunostimulatory Nanomedicines Synergize With Checkpoint Blockade Immunotherapy to Eradicate Colorectal Tumors. Nat Commun (2019) 10(1):1899. doi: 10.1038/s41467-019-09221-x 31015397 PMC6478897

[59] Xu J Xu L Wang C Yang R Zhuang Q Han X. Near-Infrared-Triggered Photodynamic Therapy With Multitasking Upconversion Nanoparticles in Combination With Checkpoint Blockade for Immunotherapy of Colorectal Cancer. ACS Nano (2017) 11(5):4463–74. doi: 10.1021/acsnano.7b00715 28362496

[60] Tanoue T Morita S Plichta DR Skelly AN Suda W Sugiura Y. A Defined Commensal Consortium Elicits CD8 T Cells and Anti-Cancer Immunity. Nature (2019) 565(7741):600–5. doi: 10.1038/s41586-019-0878-z 30675064

[61] Mager LF Burkhard R Pett N Cooke N Brown K Ramay H. Microbiome-Derived Inosine Modulates Response to Checkpoint Inhibitor Immunotherapy. Science (2020) 369(6510):1481–9. doi: 10.1126/science.abc3421 32792462

[62] Baruch EN Youngster I Ben-Betzalel G Ortenberg R Lahat A Katz L. Fecal Microbiota Transplant Promotes Response in Immunotherapy-Refractory Melanoma Patients. Science (2021) 371(6529):602–9. doi: 10.1126/science.abb5920 33303685

[63] Davar D Dzutsev AK McCulloch JA Rodrigues RR Chauvin JM Morrison RM. Fecal Microbiota Transplant Overcomes Resistance to Anti-PD-1 Therapy in Melanoma Patients. Science (2021) 371(6529):595–602. doi: 10.1126/science.abf3363 33542131 PMC8097968

